# Polarized Trafficking of AQP2 Revealed in Three Dimensional Epithelial Culture

**DOI:** 10.1371/journal.pone.0131719

**Published:** 2015-07-06

**Authors:** William L. Rice, Wei Li, Fahmy Mamuya, Mary McKee, Teodor G. Păunescu, Hua A. Jenny Lu

**Affiliations:** Center for Systems Biology, Program in Membrane Biology, Division of Nephrology, Department of Medicine, Massachusetts General Hospital and Harvard Medical School, Boston, MA 02114, United States of America; Aarhus University, DENMARK

## Abstract

In renal collecting duct (CD) principal cells (PCs), vasopressin (VP) acts through its receptor, V2R, to increase intracellular cAMP leading to phosphorylation and apical membrane accumulation of the water channel aquaporin 2 (AQP2). The trafficking and function of basolaterally located AQP2 is, however, poorly understood. Here we report the successful application of a 3-dimensional Madin-Darby canine kidney (MDCK) epithelial model to study polarized AQP2 trafficking. This model recapitulates the luminal architecture of the CD and bi-polarized distribution of AQP2 as seen in kidney. Without stimulation, AQP2 is located in the subapical and basolateral regions. Treatment with VP, forskolin (FK), or 8-(4-Chlorophenylthio)-2′-O-methyladenosine 3′,5′-cyclic monophosphate monosodium hydrate (CPT-cAMP) leads to translocation of cytosolic AQP2 to the apical membrane, but not to the basolateral membrane. Treating cells with methyl-β-cyclodextrin (mβCD) to acutely block endocytosis causes accumulation of AQP2 on the basolateral membrane, but not on the apical membrane. Our data suggest that AQP2 may traffic differently at the apical and basolateral domains in this 3D epithelial model. In addition, application of a panel of phosphorylation specific AQP2 antibodies reveals the polarized, subcellular localization of differentially phosphorylated AQP2 at S256, S261, S264 and S269 in the 3D culture model, which is consistent with observations made in the CDs of VP treated animals, suggesting the preservation of phosphorylation dependent regulatory mechanism of AQP2 trafficking in this model. Therefore we have established a 3D culture model for the study of trafficking and regulation of both the apical and basolaterally targeted AQP2. The new model will enable further characterization of the complex mechanism regulating bi-polarized trafficking of AQP2 in vitro.

## Introduction

In the mammalian kidney, water reabsorption is regulated by vasopressin (VP), which stimulates the membrane accumulation of aquaporin 2 (AQP2) resulting in an increased water permeability of the apical plasma membrane of CD principal cells. Upon binding to its receptor, V2R, VP causes an increase of intracellular cAMP, subsequent phosphorylation of AQP2 and redistribution of AQP2 from cytoplasmic vesicles to the apical plasma membrane, thus allowing water transport to occur [1]. The VP-V2R-AQP2 axis is critical to the maintenance of water balance and its dysfunction leads to diabetes insipidus and water/ electrolyte imbalance as seen in congestive heart failure and cirrhosis [2–5].

AQP2 is widely considered to be an apically located membrane channel that responds to VP regulation both in vitro and in vivo [6,7]. However, it has also been observed that AQP2 has a bi-polarized distribution in the kidney collecting duct, with both basolateral and apical/subapical expression. However, the physiological function of basolaterally located AQP2 is not yet understood [8]. A few studies have reported that basolateral AQP2 can be modified by oxytocin, aldosterone and hypertonicity in animals [9–11]. In vitro, hypertonicity was found to induce AQP2 redistribution to basolateral membranes [11] and our group has revealed that a "cold shock" of 4°C causes basolateral membrane accumulation of AQP2 in MDCK cells grown on a permeable filter [12]. Recently, we have reported that through interaction with integrin β1, which is prominently located on the basal membrane of the CD of the kidneys, AQP2 modulates the trafficking of integrin β1, thus demonstrating a novel role for this water channel in mediating cell migration and epithelial morphogenesis [13]. In addition, integrin signaling and integrin linked kinase have been shown to regulate AQP2 trafficking, supporting a possible link between extracellular matrix-integrin signaling and AQP2 trafficking [14,15]. These observations prompted us to revisit the function and regulation of the trafficking of basolaterally located AQP2.

Phosphorylation plays a key regulatory role in AQP2 trafficking. In addition to phosphorylation of AQP2 at its key c-terminal residue, serine 256, it has been shown that VP also modulates the phosphorylation of additional serine residues (S261, S264, S269) residing on the C terminus of AQP2. The phosphorylation of the C terminal serines critically influences the association of AQP2 with key components of the trafficking machinery such as the 70 kilodalton heat shock proteins (HSP70), dynamin [16,17] as well as the membrane accumulation of AQP2 [18–20]. Thus far, the role of AQP2 phosphorylation in AQP2 trafficking has been investigated in animal models such as the VP deficient Brattleboro rat, and the *Xenopus* oocyte [6,21,22] along with various cell culture models expressing AQP2 phosphorylation mimics (substitution of alanine (A), or aspartic acid (D) for the serine) [7,18–20,23–25]. Studying the spatial distribution of differentially phosphorylated AQP2 and understanding the contribution of phosphorylation of AQP2 to its polarized apical versus basolateral membrane trafficking is important for further dissecting the regulatory mechanism of polarized AQP2 trafficking in vitro and in vivo.

To address these challenges, we have established a well-defined three dimensional epithelial culture model using MDCK cells that has been previously applied to study epithelial processes including "cyst" formation, tubulo-morphogenesis, ciliogenesis and epithelial polarity [26–29]. Here, we report that this 3D culture model recapitulates the tissue architecture of the collecting duct, enables the study of the trafficking and regulation of basolaterally versus apically targeted AQP2, as well as, for the first time in vitro, the observation of the polarized distribution of differentially phosphorylated forms of AQP2.

## Results and Discussion

### AQP2 expressing MDCK cells form polarized 3D epithelial cysts

**Fig 1:**
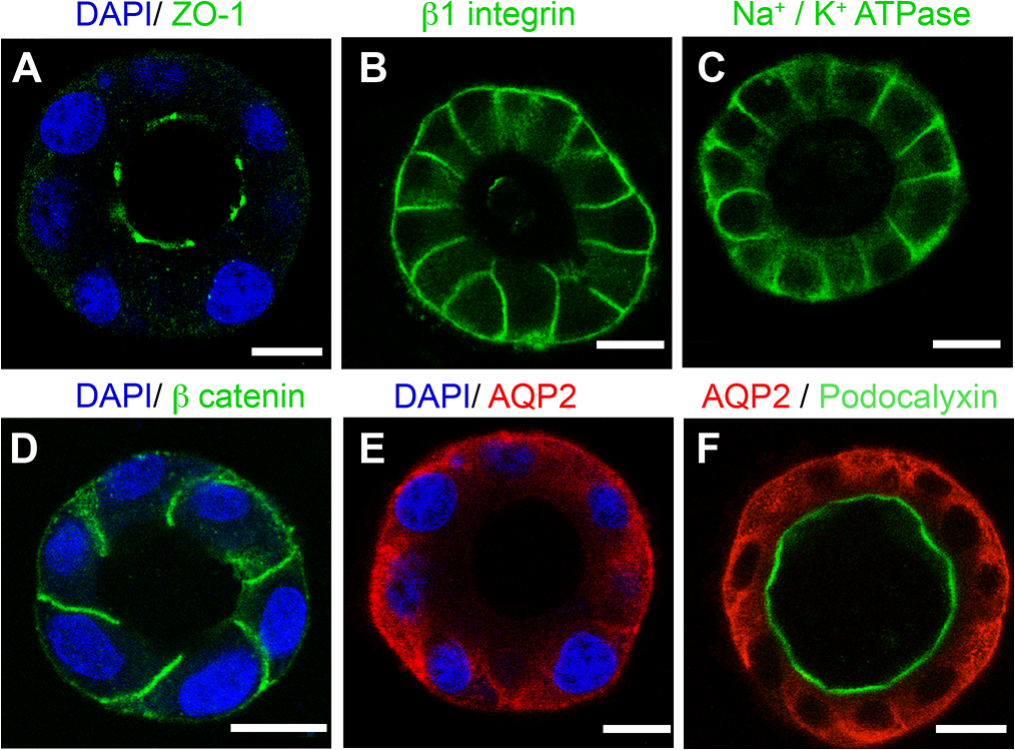
Formation of well-polarized epithelial cyst by AQP2 expressing MDCK cells. Single MDCK cells expressing rat AQP2 plated on matrigel form well-polarized cyst. The apical membrane domain is ringed by the tight junction protein (A) Zona Occludens-1 (green) and highlighted by (F) podocalyxin (green). (B) Integrin β1, (C) Na+/K+-ATPase and (D) β-catenin (green) are located on the basolateral membrane. (E, F), AQP2 (red) is distributed diffusely in the cytosol and in the basolateral region under baseline, non-stimulated conditions. DAPI (blue) stains nuclei. Images are single confocal planes taken through the middle of a spherical cyst. Bars = 10 μm.

AQP2 expressing MDCK cells form polarized spherical cyst structures after 5–7 days of culture on matrigel (Fig 1 and S1 Fig). The tissue architecture of the”cyst” closely resembles the mature collecting duct epithelium observed *in vivo*: it is polarized with an outward facing basal membrane in contact with the extracellular matrix (ECM), lateral membranes that connect neighboring cells and a free apical membrane facing a fluid filled lumen. This polarized epithelium mimic expresses well-defined epithelial polarity markers: the classic apical markers GP-135 (podocalyxin) and zona occludens -1 are clearly detected on the apical membrane (Fig 1A and 1F respectively), while basolateral proteins such as integrin β1, Na+/K+-ATPase and β-catenin are restricted to the basolateral domain of the cyst (Fig 1B, 1C and 1D respectively). These results are consistent with other reports using similar models and observations of epithelium *in vivo* [30–33]. In the cyst, AQP2 locates mainly in vesicles throughout the cytosol and in the basolateral membrane region under baseline, non-stimulated conditions (Fig 1E and 1F), similar to what is observed in the connecting tubule and inner medullary collecting duct in normal rats [34].

**Fig 2:**
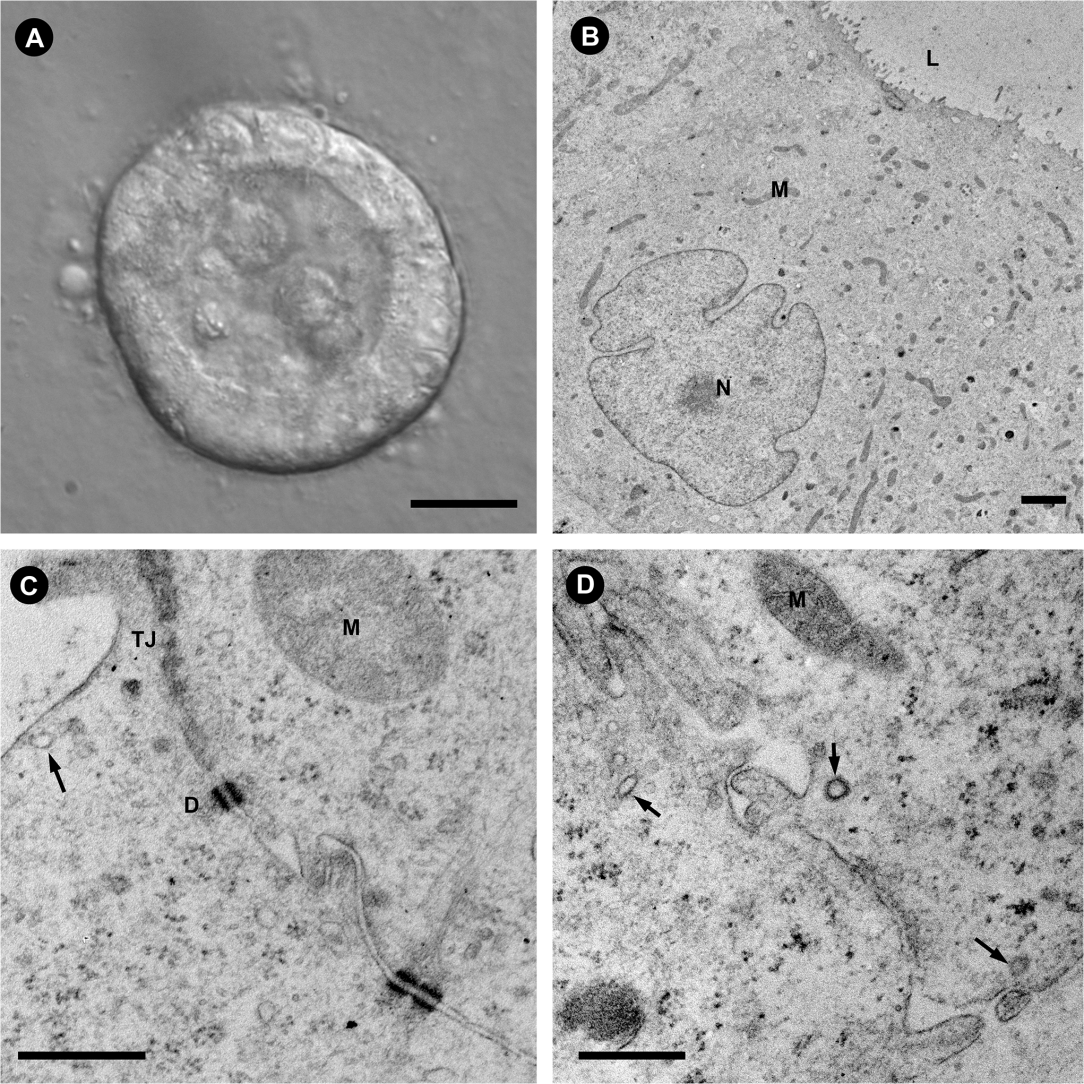
Ultrastructure of MDCK-AQP2 cysts. The ultrastructure of the cyst closely resembles the polarized architecture of renal tubular epithelium in animal kidney. (A) A differential interference contrast image of a whole cyst. The lumen is clearly visible despite some remnants of cells that were shed in the lumen during lumen formation. The basal membrane is in contact with the Matrigel substrate. Scale = 10 μm. (B) In a TEM cross section of a single cell from a MDCK-AQP2 cyst, microvilli can be seen on the apical membrane facing the lumen (L) while the nucleus (N) is oriented toward the basal membrane. Scale = 2 μm. (C) At the apical domain, neighboring cells interact through tight junctions (TJ) and desmosomes (D) and the presence of clathrin-coated pits (arrow) indicate active endocytosis in the apical and subapical region. Scale = 500 nm. (D) Similarly, clathrin coated vesicles are observed at the basal and lateral membranes of the cyst cells (arrows). Scale = 500 nm. In all images (M) indicates mitochondria.

The ultrastructure of the MDCK cell cysts was examined by transmission electron microscopy (TEM), as shown in Fig 2. Multiple microvilli are observed on the apical membrane facing the lumen while the nuclei are oriented toward the basal membrane (Fig 2B). The presence of tight junctions (TJ) on the apical pole of the lateral membranes between two adjacent cells is clearly revealed by the EM (TJ in Fig 2C). Multiple desmosomes (D in Fig 2C) are clearly visualized along the lateral membrane below the apical pole/TJ. Clathrin coated vesicles (arrows) are visible in both the apical and basolateral regions of the cells, suggesting the presence of an active endocytic trafficking interface on both the apical and basolateral membranes.

Emerging observations suggest a possible important function of basolateral AQP2 in collecting duct development [13], prompting a search for a new in vitro model in which to study basolaterally enriched AQP2. Generally, membrane protein trafficking models utilizing cells grown on glass or tissue culture plastic, are not well polarized and AQP2 is usually found in the perinuclear region. Upon stimulation by VP, AQP2 often traffics to the basolateral membrane instead of the apical membrane as observed *in vivo* [35,36]. When grown on permeable filter supports, MDCK cells form a polarized monolayer, exhibit common polarity markers, and AQP2 traffics to the apical membrane in response to FK or VP [37–39]. Despite the detection of basolateral AQP2 under chronic hypertonic treatment or acute cold block at 4°C in filter grown MDCK cells [11,12], basolateral localization of AQP2 under baseline, non-stimulated conditions, as occurs *in vivo* in parts of the collecting duct system [11,34] has not been observed. Therefore our 3D epithelial culture model represents a unique model for studying bi-polarized, basolateral and apical AQP2 trafficking in the context of a well-defined polarized epithelium structure and contains more favorable cell-ECM and cell-cell interaction and signaling resembling that found in kidneys in vivo.

### Polarized trafficking of AQP2 in the MDCK-AQP2 3D culture

**Fig 3:**
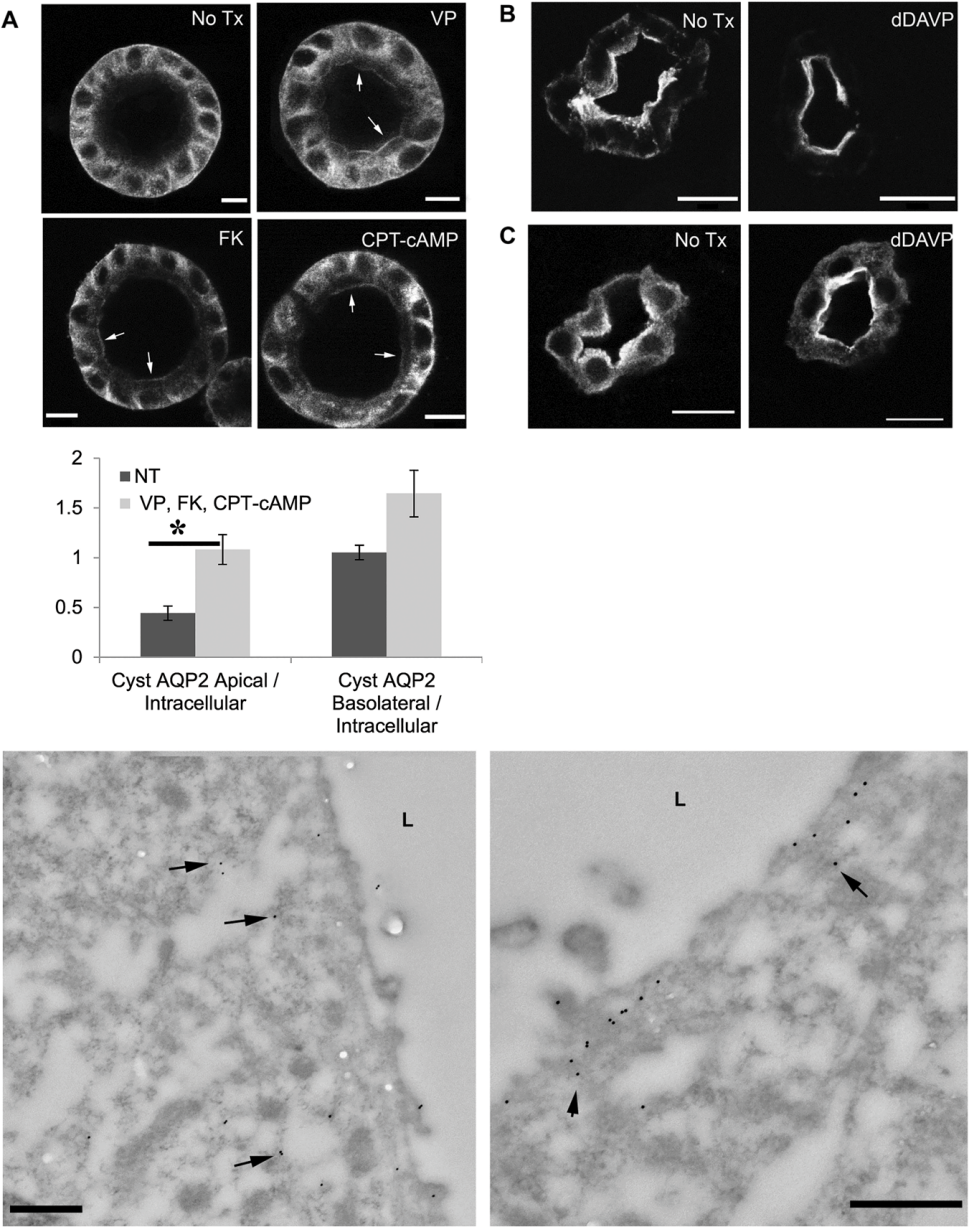
Regulated trafficking of AQP2 is intact in MDCK cysts. AQP2 trafficking in MDCK-AQP2 cysts is intact and staining patterns for total AQP2 are comparable to those observed in Brattleboro, and normal rat kidney. (A) MDCK-AQP2 cysts were incubated in serum free medium for 120 minutes. Addition of AVP, FK, or CPT-cAMP to the medium for 40 minutes resulting in apical membrane accumulation of AQP2 (arrows). Bar = 10 μm. (D) Asterisk denotes that significantly (P < = 0.05) more apical, but not basolateral staining of AQP2, relative to intracellular AQP2, was observed following stimulation with AVP, FK or CPT-cAMP. N = 5 cysts (NT, non-treated), N = 12 cysts (AVP/FK/CPT-cAMP stimulated). The data for AVP, FK and cAMP-treated cysts were pooled together because we saw no statistically significant difference in Apical/Internal total AQP2 or Basolateral/Internal total AQP2 between the treatment modalities. (B) In the Brattleboro rat kidney AQP2 was located mainly in the subapical region while apical membrane accumulation of AQP2 was seen after treatment with dDAVP for 3 days. Bar = 10 μm. (C) Similarly, in a tissue slice culture from normal rat kidney, incubation in medium without VP resulted in AQP2 in the cytosol and subapical region. dDAVP treatment for 20 minutes resulted in AQP2 translocation to the apical membrane, with AQP2 still detectable in the cytosol. (E) In transmission electron micrographs, AQP2 in the MDCK-AQP2 cyst is labeled with 15nm gold particles. AQP2 gold particles distributed diffusely throughout the cytosol under baseline, non-stimulated conditions (left panel) while AQP2 accumulated on the apical membrane after VP stimulation (right panel), but not on the basolateral membrane (S2 Fig). Bars = 500 nm

To demonstrate the utility of this model to study polarized trafficking of AQP2, the epithelial “cysts” were treated with arginine vasopressin (AVP), forskolin (FK) CPT-cAMP, or m0βCD. Application of AVP, FK or CPT-cAMP, leads to apical accumulation of AQP2 (Fig 3A) suggesting an intact, regulated trafficking pathway of AQP2 at the apical and subapical domain in cells that form the cyst. While clearly weaker than the apical accumulation of total AQP2 seen in vivo, this data is consistent with our observations in the Brattleboro (Fig 3B) [40] and normal (Fig 3C) rat kidney, in which a similar apical redistribution of AQP2 is detected after 1-desamino-8-D-arginine vasopressin (dDAVP) treatment. The apical trafficking of AQP2 in the cysts was further confirmed by immunogold electron microscopy (Fig 3E). Under non-stimulated conditions, AQP2 is found in the cytosol and subapical domain while a clear apical accumulation of AQP2 occurs after VP treatment. Despite the clear apical membrane redistribution of total AQP2 in response to AVP, FK or CPT-cAMP, there was no significant enrichment of AQP2 signal on the basolateral membrane in response to these treatments (Fig 3D, S2 Fig), suggesting that AQP2 located near/at the basolateral region may not be readily subject to VP/cAMP regulation as is the apically located protein.

**Fig 4:**
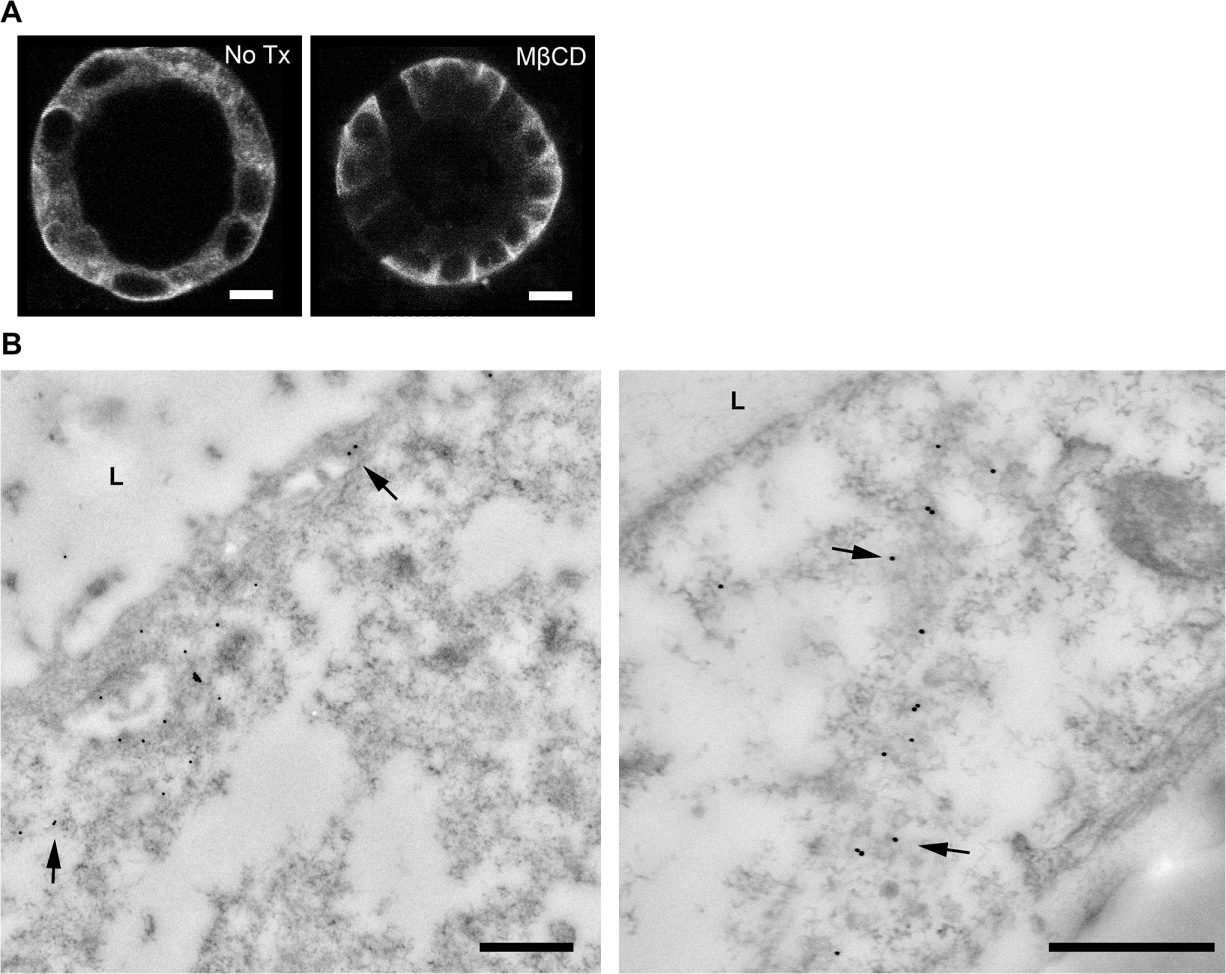
Constitutive recycling of AQP2 is detected in the basolateral domain of the MDCK-AQP2 cyst. In the MDCK-AQP2 cyst, blockade of endocytosis by addition of mβCD for 20 minutes led to predominant enrichment of AQP2 in the basolateral domain. (A) Immunofluorescence staining reveals basolateral accumulation of total AQP2 with mβCD treatment. (B) In transmission electron micrographs, some 15nm gold AQP2-labeled particles are found in the apical/subapical domain (left panel) but they are more predominantly presented on the basolateral membranes of two neighboring cells. Bar = 500 nm

Previously, using a less well-polarized cell culture model [41], we have shown that, in addition to regulated trafficking, AQP2 is also constitutively recycling inside cells. Acute or chronic blockade of endocytosis by mβCD in these cells causes membrane accumulation of AQP2 by inhibiting endocytosis. In the 3D epithelial model, treatment with mβCD for 20 minutes results in accumulation of AQP2 in the basolateral membrane, without detectable accumulation of total AQP2 signal in the apical membrane by immunofluorescence staining (Fig 4A). Increased accumulation of AQP2 in the basolateral membrane after treatment with mβ-CD is further revealed by immunogold electron microscopy (Fig 4B). This data suggests that active insertion and endocytotic removal of AQP2 occurs in the basolateral domain of polarized MDCK cells grown in the 3D culture which is consistent with our observation of active clathrin coated vesicles in the basolateral domain of the cyst and with our recent observations in MDCK cells grown on filters [12]. The lack of observed apical membrane accumulation of AQP2 in mβCD treated cysts may be due to the fact that mβCD first encounters the basolateral membranes of the cysts followed later by the organelle and apical membrane dmains. Thus AQP2 is probably sequestered in the basolateral domain (and is unavailable for further trafficking) while apical endocytosis is still ongoing to deplete the apical domain of the remaining AQP2.

Another possibility is that a transcytosis pathway mediates AQP2 translocation from the basolateral domain to the apical membrane. Therefore, blocking endocytosis of AQP2 on the basolateral domain would subsequently affect the transcytotic transport of AQP2 to the apical membrane domain. AQP2 transcytosis has been hinted at by previous observations [11,42] and is further suggested by a recent report from our group [12]. We have shown that 4°C cold shock of MDCK cells causes AQP2 accumulation on the basolateral membrane, and this basolateral AQP2 undergoes rapid internalization and transcytosis to the apical membrane upon VP/FK stimulation after rewarming. This intriguing observation of transcytosis of the "cold shock" induced basolateral AQP2 needs to be further examined.

### Polarized trafficking of phosphorylated AQP2 in the MDCK-AQP2 3D culture

**Fig 5:**
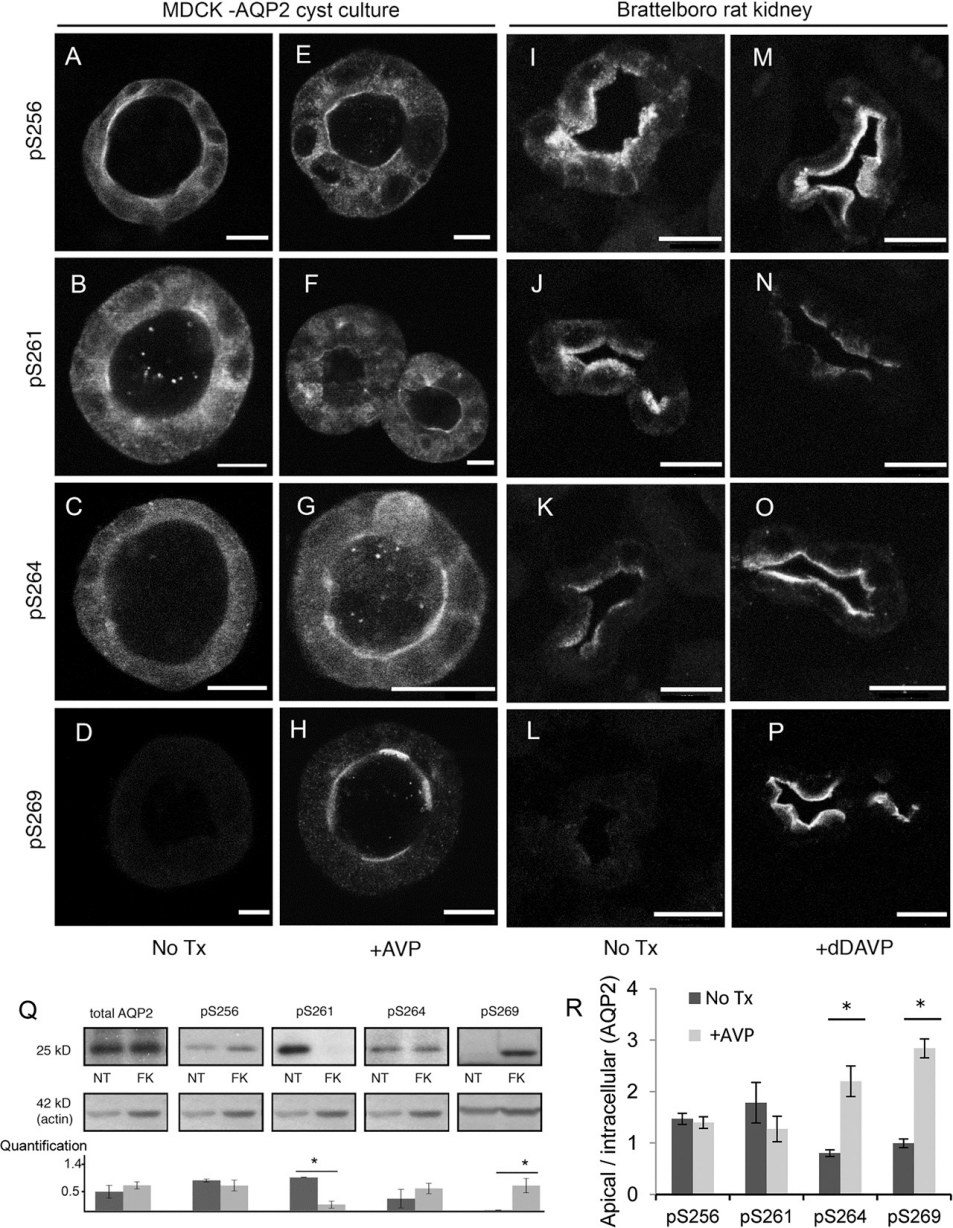
Subcellular distribution of phosphorylated AQP2 in MDCK cells grown in 3D culture and in Brattleboro rat kidney. Antibodies recognizing AQP2 phosphorylated at S256, S261, S264 or S269 highlighted the subcellular localization of phosphorylated AQP2 in MDCK-AQP2 cysts (A-H). The staining pattern of differentially phosphorylated AQP2 in the cyst is reminiscent of that observed in Brattleboro rat kidneys (I-P). Prior to stimulation, cysts were pre-incubated in serum free medium for 120 min. Comparing the distribution of phosphorylated AQP2 in cyst versus kidney tissue, pS256 is found in the cytosol and apical membrane in both the cyst and kidney tissue without VP treatment (A, I) Total AQP2 staining of the tissue in panel I can be seen in Fig 3B. After stimulation with VP (E,M), the redistribution of pS256 AQP2 to the apical membrane is pronounced in the kidney tissue, but is not significantly different in the cyst tissue (quantification in panel R). VP stimulation resulted in a reduction of both apical and cytosolic pS261 fluorescence intensity in cyst and kidney tissue (B vs. F, J vs. N). Under stimulated conditions, some non-specific labeling of the nuclei by the p261 antibody can be observed (F). pS264 staining signal translocated from the cytosolic compartment (C,K) to the apical membrane (G, O) after VP stimulation in both cyst and kidney tissue (H, P). In both the cyst, and the rat tissue pS269 staining was only observed following stimulation, and solely located on the apical membrane (H,P). Bar = 10 μm. (Q) Differential phosphorylation of AQP2 at various serine residues after FK stimulation was detected by western blot in AQP2 expressing MDCK cells grown as a monolayer culture. The bar graph represents quantification of western blots (means ± SE, N = 3 experiments): total AQP2 and phosphorylation antibody results are presented as relative to loading control. No significant difference was observed between non-stimulated and VP/FK simulated conditions for AQP2 pS256 or pS264. On the other hand, stimulation resulted in a significant (asterisk) decrease in pS261 (p = .002) and a significant increase in pS269 (p = .04). (R) Quantitative assessment of cyst immunofluorescence images reveal significant (P < = 0.05) increases in apical, relative to intracellular, pS264 (N = 4 cysts) and pS269 (N = 8 cysts) AQP2 staining following AVP stimulation. No significant difference in apical, relative to intracellular pS261 (N = 5 cysts) fluorescence is observed due to concomitant decreases (relative to treatment) in both apical and intracellular pS261 staining.

We next examined the phosphorylation of AQP2 and its trafficking in the polarized epithelial cyst model. Immunostaining using antibodies specific for AQP2 phosphorylation at S256, S261, S264, or S269 in the absence (Fig 5A–5D) or presence (Fig 5E–5H) of VP treatment reveals that all four phosphorylation specific antibodies are able to recognize AQP2 in the 3D epithelial culture and that the staining patterns of individually phosphorylated AQP2 are reminiscent of what was observed in the dDAVP treated Brattleboro rat kidneys (Fig 5I–5L no treatment, and Fig 5M–5P dDAVP treated).

Under baseline conditions, AQP2 phosphorylated at S256 (pS256) is found throughout the cell including some presence on the basolateral and apical membrane (Fig 5A). While apical pS256 AQP2 is observed following VP stimulation (Fig 5E), it is not significantly different than that observed under non-stimulated conditions (Fig 5R). In kidneys from VP deficient Brattleboro rats, pS256 AQP2 can be seen throughout the cytoplasm (Fig 5I) with increased apical staining upon treatment by VP (Fig 5M). There is detectable baseline staining of pS256 AQP2 in our 3D culture model and in VP treated Brattleboro rats suggesting a baseline activation of pS256 AQP2 in vitro and in vivo. The baseline phosphorylation of S256 AQP2 is confirmed by western blot, and there is no dramatic increase in the total amount of pS256 with maximal stimulation by VP/FK (Fig 5Q), a phenomenon that has been described by others [43,44].

VP stimulation leads to a decrease in pS261 AQP2, possibly through an as-yet-unknown phosphatase. Without VP treatment, pS261 is observed throughout the cytoplasm with some apical localization (Fig 5B). Upon VP stimulation (Fig 5F), the overall pS261 AQP2 intensity decreases and the remaining pS261 staining is more prominent on the apical membrane. A similar reduction of the overall pS261 AQP2 staining and slight enrichment of the apical staining of pS261 AQP2 is also observed in VP treated Brattleboro rat kidneys (Fig 5J vs. 5N), and is consistent with published reports [25,45]. The VP induced reduction of total pS261 AQP2 is confirmed by western blot (Fig 5Q).

Overall, the greatest agreement in AQP2 phosphorylation and distribution between our 3D culture and animal data are observed from S264 and S269 AQP2. VP stimulation causes significant apical accumulation of pS264 and pS269 AQP2 signal (Fig 5G and 5H) compared to untreated cysts (Fig 5C and 5D) respectively, similar to what has been reported in the kidneys of VP treated Brattleboro rat (Fig 5K and 5O, and 5L and 5P). The level of total pS264 AQP2 is not significantly increased by VP/FK by western blot (Fig 5Q), suggesting that VP/FK treatment may not alter the level of pS264 AQP2, but rather regulates the apical redistribution of pS264 AQP2 (Fig 5R). In contrast, VP/FK causes a dramatic increase in the total pS269 AQP2 compared to a non-detectable level of pS269 AQP2 at baseline by western blot (Fig 5Q), which is clearly seen on the apical membrane in VP treated cysts (Fig 5H and 5R) and in rats (Fig 5P). It suggests that phosphorylated S269 AQP2 only occurs on the plasma membrane [20], and is in agreement with a previous report [44]. Therefore, our data suggests that the phosphorylation dependent regulation of AQP2 trafficking observed in vivo is preserved in this epithelial 3D culture model. This new model can thus be used to elucidate the role of phosphorylation of each serine residue in the polarized trafficking of AQP2 in vivo.

In summary, our study presents a novel application of a three-dimensional epithelial culture model for studying the bi-polarized trafficking of AQP2 in vitro. It suggests the presence of possible differential trafficking pathways for apical versus basolateral AQP2 versus a transcytotic pathway of AQP2, and likely preserved phosphorylation dependent regulatory mechanism in this 3D epithelial model. More importantly, it provides a unique in vitro system to study the poorly understood trafficking and regulation of basolaterally targeted AQP2 in vitro and will prompt further comprehensive investigation of polarized trafficking of AQP2.

## Materials and Methods

### Chemicals, reagents and antibodies

Unless otherwise noted chemical reagents were purchased from Sigma-Aldrich (St. Louis MO). Cell culture medium was purchased from Invitrogen (Grand Island NY). The expression of total AQP2 in cells was assessed with a polyclonal goat antibody raised against the C terminus of AQP2 that was purchased from Santa Cruz Biotechnology (Santa Cruz, CA) (sc-9882). Rabbit antibodies recognizing phosphorylation of the serine residues at the 256, 261,264 or 269 position of AQP2 were gifts from Dr. Mark Knepper at NIH and purchased from PhosphoSolutions (Aurora, CO). Mouse antibodies against the apical glycoprotein GP-135, were a gift from Dr. George Ojakian [30]. Anti-Beta catenin was from Santa Cruz (sc-31001 E-17) and anti-ZO-1 is a gift from Dr. Eveline Schneeberger (MGH).

### Cell culture/formation of a cyst like structure

MDCK cells, were stably transfected with rat AQP2 as previously described [38], and maintained in DMEM (Invitrogen) supplemented with 7% FCS and 1% penicillin-streptomycin under a humidified atmosphere with 5% CO_2_ at 37°C. The procedure of forming the cyst was previously described [46]. Briefly, Labtek II 8 well chamber slides were coated with Matrigel (BD). A single cell suspension of 2,000 MDCK cells mixed with 2% v/v Matrigel was placed in each well. Cysts with visible lumens were apparent by the 5th to 7th day of incubation.

### AQP2 trafficking

Fully formed cysts were washed and incubated in serum free medium 120 min prior to treatment. To stimulate the trafficking of AQP2, 1x10^-8^ M AVP and/or 1x10^-5^ M FK or CPT-cAMP (8-(4-Chlorophenylthio)-2′-O-methyladenosine 3′,5′-cyclic monophosphate monosodium hydrate) was added to the medium for 30 minutes. For “cysts” treated with CPT-cAMP, a preincubation with 3-isobutyl-1-methylxanthine (IBMX) (Sigma Aldrich I7018) at a final concentration of 1mM for 30 min was performed prior to adding cAMP (Sigma Aldrich C8988). Treating cells with VP and FK alone or in combination gives similar result.

### Animals

Animal experiments were approved by Institutional Committee on Research Animal Care, in accordance with National Institutes of Health (NIH) *Guide for the Care and Use of Laboratory Animals*. Briefly, dDAVP was delivered to adult male Brattleboro rats via an Alzet osmotic pump that was implanted into the subcutaneous tissue at the nape of the neck of the animal as previously reported [40,47]. Three days after treatment, animal kidneys were fixed by perfusion-fixation with PLP (paraformaldehyde (4%) lysine (10 mM) periodate (10 mM) (PLP) in 5% sucrose, 0.1 mM sodium phosphate) and processed for immunostaining as previously reported [48].

For in vitro kidney slice experiments, kidney slices were prepared as described previously [49]. Briefly, adult Sprague-Dawley rats were anesthetized using isofluorane. Kidneys were harvested and slices of approximately 0.5 mm were cut using a Stadie-Riggs microtome. All of the sliced kidneys were incubated at 37°C for 15 min in equilibrated Hank’s balanced salt solution (HBSS) (pH 7.4, with 5% CO_2_). After equilibration, the slices were incubated in HBSS containing chemicals (100 μM AVP and 100 μM FK, 40 μM AG-490, 50 μM β-lapachone, or 0.1% DMSO as control) for 30 min. After incubation, all of the samples were immersed in 4% PLP fixative.

### Immunofluorescence staining and immunoblot

Immunofluorescence staining of cysts was performed as following: After fixation with 4% paraformaldehyde in PBS for 20 minutes, “cysts” were washed three times for 5 min in PBS and permeabilized with 0.1% w/v Saponin in 0.7% gelatin solution for 20 minutes. After permeabilization, “cysts” were incubated overnight with primary antibodies at 4°C, and then with fluorescent secondary antibodies. Immunofluorescence staining of kidney tissues was performed as previously reported [48]. Briefly, after perfusion-fixation, kidneys were cryosectioned into 5 μm thick sections and permeabilized with 0.1% SDS for 4 minutes. The kidney sections were incubated with primary antibodies at 4°C overnight followed by staining with fluorescent secondary antibodies. Finally, immunostained “cysts” or kidney section were visualized using a Bio-Rad Radiance 2000 confocal laser scanning microscope with a 20x (NA 0.75) or 40x (NA 1.0) objective or on a Nikon AR-1 confocal laser scanning microscope. Immunobloting was performed as previously described without modification [16]. Immunoblot quantification was performed using the built in gel analysis tool in ImageJ (1.42), briefly, lanes were selected by equal sized rectangular regions of interest, the profiles of these regions were plotted and the area under the curve was taken to be the band density. In Fig 5Q the density is displayed as relative to loading control (Actin or Gapdh). The fluorescence intensity of the apical and basolateral membranes in cyst immunofluorescence images were quantified relative to the intracellular fluorescence intensity in ImageJ (1.42). Briefly, the mean fluorescence intensity was measured from the apical membrane, basolateral membrane and intracellular space (from the same cells within a cyst) using a region of interest defined by a 20 pixel wide line tool.

Significance for differences in means (with a p value less than 0.05) were tested using the Students T test in Excel (Microsoft, Redmond, WA). The results of quantifications are represented as means with standard error in Figs 3 and 5.

### Transmission Electron Microscopy

MDCK-AQP2 cells were plated on Matrigel coated permeable filters and allowed to form “cysts”. For ultrastructural analysis “cysts” were fixed in 2.0% glutaraldehyde in 0.1 M sodium cacodylate buffer, pH 7.4 (Electron Microscopy Sciences, Hatfield, PA) overnight at 4 C, then post-fixed in 1.0% osmium tetroxide in cacodylate buffer for one hour at room temperature, followed by dehydrated through a graded series of ethanol to 100%. They were then infiltrated with Epon resin (Ted Pella, Redding, CA) in a 1:1 solution of Epon:ethanol and embedded in Epon at 60°C. Thin sections were cut on a Leica EM UC7 ultramicrotome, collected onto formvar-coated grids, stained with uranyl acetate and lead citrate. For immunogold electron microscopy, the “cysts” were fixed in 4% paraformaldehyde (EMS) for one hour at room temperature. After dehydration through a graded series of ethanol, they were embedded in LR white resin (EMS). Thin sections were cut, incubated on drops of primary antibody for one hour at room temperature, then rinsed on drops of PBS, followed by incubation on drops of IgG gold for one hour. They were rinsed on drops of double deionized water (DDH_2_O) and stained with 2.0% uranyl acetate for 5 min and rinsed with DDH_2_O. All grids were examined in a JEOL JEM 1011 transmission electron microscope at 80 kV. Images were collected using an AMT digital imaging system (Advanced Microscopy Techniques, Danvers, MA). Structures in TEM images were identified by their morphology, structure, electron density and cellular localization. Post-fixation renders the clathrin coat electron dense (i.e., dark) and this appearance distinguishes clathrin-coated vesicles from other kinds of vesicles. Clathrin coated vesicles are electron dense membrane-bound annuli, approximately 120–150 nm in diameter and appear as having a thicker membrane. Mitochondria are more electron dense than the surrounding cytosol, have a characteristic double membrane, are of an elliptical shape and can vary in size from approximately 500 nm to sizes on the order of μm. On higher magnification images such as Fig 2C and 2D the folded morphology of the internal mitochondrial membrane (cristae) is visible. Desmosomes appear as electron dense structures on each side of adjacent basolateral membranes with an electron-light band appearing at the interface of each structure. Tight junctions can be described as “fuzzy” electron dense clusters at interfaces between two adjacent cellular membranes that appear to be pinched together.

## Supporting Information

S1 Fig MDCK Cyst structure is spherical. A volumetric rendering of one hemisphere of an MDCK cyst reveals that the cysts are spherically shaped. In this figure, 5μM thick optical sections were taken with a confocal microscope through half of an MDCK cyst stained for ZO1 (green) and actin (red). ZO1 highlights both the boundaries between individual MDCK cells, and the boundaries between the apical and basolateral membrane domains in each MDCK cell. The actin staining highlights the periphery of each individual cell.
(PNG)

S2 Fig MDCK AQP2 Immunogold. In transmission electron micrographs of AVP stimulated MDCK cysts, AQP2, highlighted by 15 nm gold spheres (arrows), is observed to accumulate in the apical membrane (left panel) while minimal basolateral AQP2 is observed from AVP treated MDCK cysts in the same embedded block. Scale = 500 nm.
(PNG)

S1 Zipfile MDCK cyst immunofluorescence images. These zipfiles contain the additional immunofluorescence images of MDCK cysts used for quantification. The included images are of stimulated and non-stimulated MDCK cysts stained with antibodies against total AQP2, as well as pS256, pS261, pS264 and pS269 phospho-AQP2.
(ZIP)

S2 Zipfile MDCK AQP2 Western bolts. These zipfiles contain the additional western blot data used for quantification from stimulated and non-stimulated MDCK cells. The blots are probed with antibodies for GAPDH, total AQP2, as well as pS256, pS261, pS264 and pS269 phospho-AQP2.
(ZIP)
