# Supplementary figures and images for: Polarized Trafficking of AQP2 Revealed in Three Dimensional Epithelial Culture
Source: PLoS One. 2015 Jul 6;10(7):e0131719. doi: 10.1371/journal.pone.0131719 (PMC4493001; doi:10.1371/journal.pone.0131719)

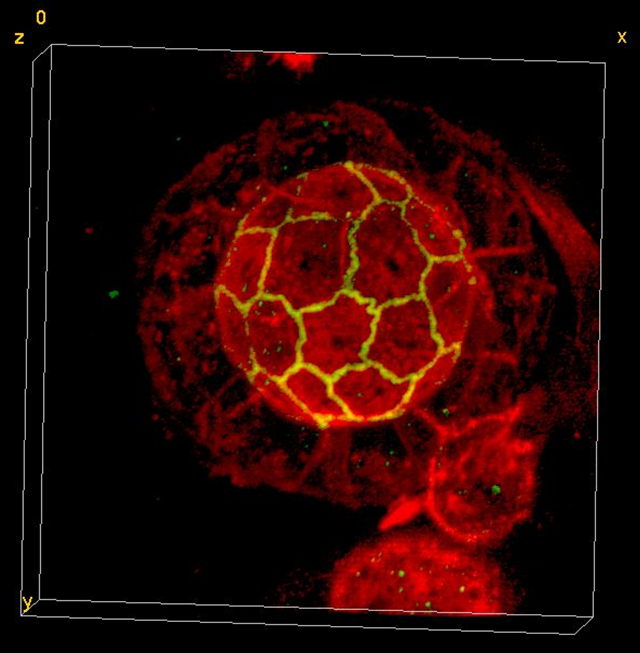

Supplement: S1 Fig — A volumetric rendering of one hemisphere of an MDCK cyst reveals that the cysts are spherically shaped. In this figure, 5μM thick optical sections were taken with a confocal microscope through half of an MDCK cyst stained for ZO1 (green) and actin (red). ZO1 highlights both the boundaries between individual MDCK cells, and the boundaries between the apical and basolateral membrane domains in each MDCK cell. The actin staining highlights the periphery of each individual cell. (PNG) [file pone.0131719.s001.PNG]

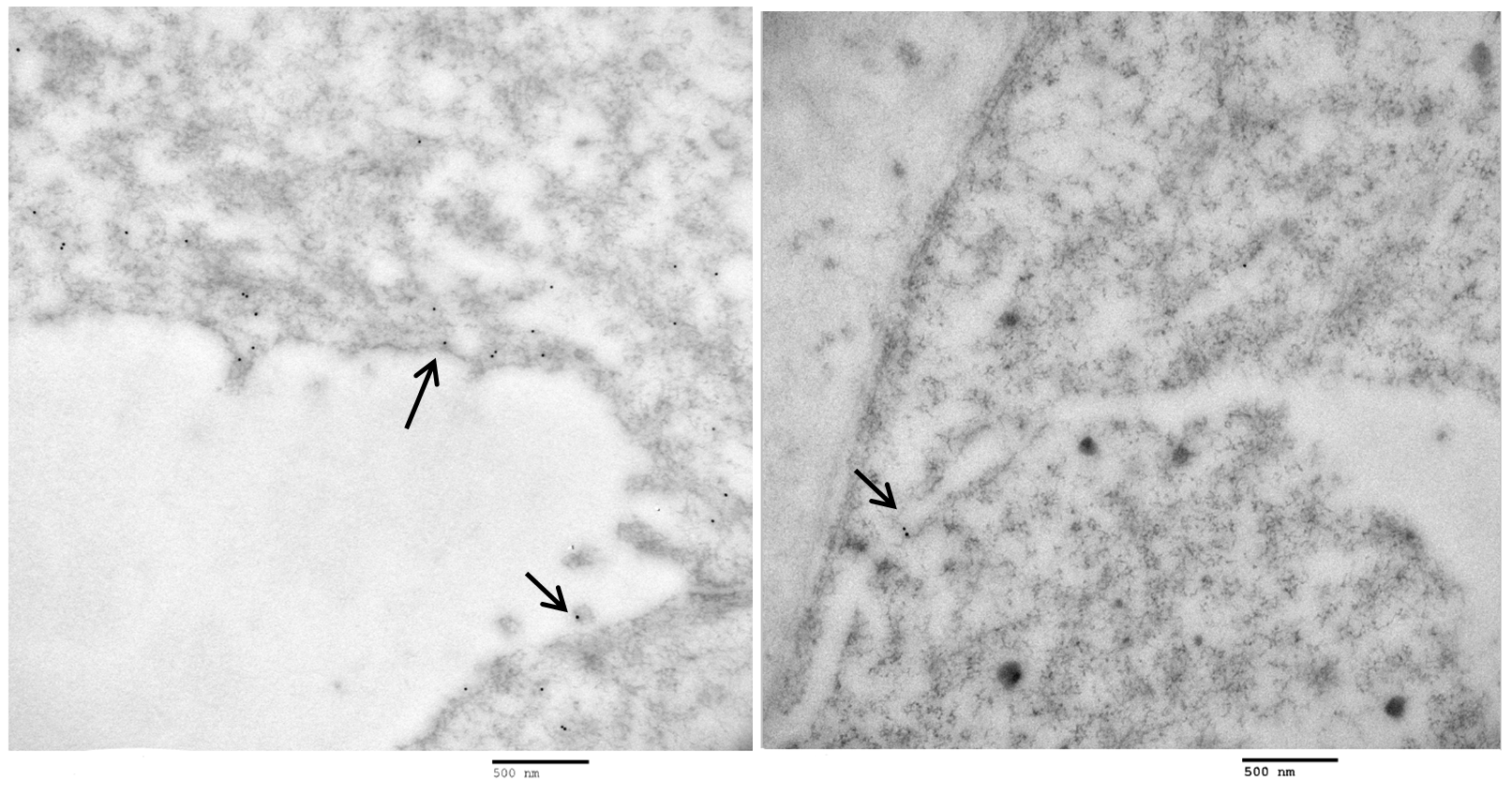

Supplement: S2 Fig — In transmission electron micrographs of AVP stimulated MDCK cysts, AQP2, highlighted by 15 nm gold spheres (arrows), is observed to accumulate in the apical membrane (left panel) while minimal basolateral AQP2 is observed from AVP treated MDCK cysts in the same embedded block. Scale = 500 nm. (PNG) [file pone.0131719.s002.PNG]

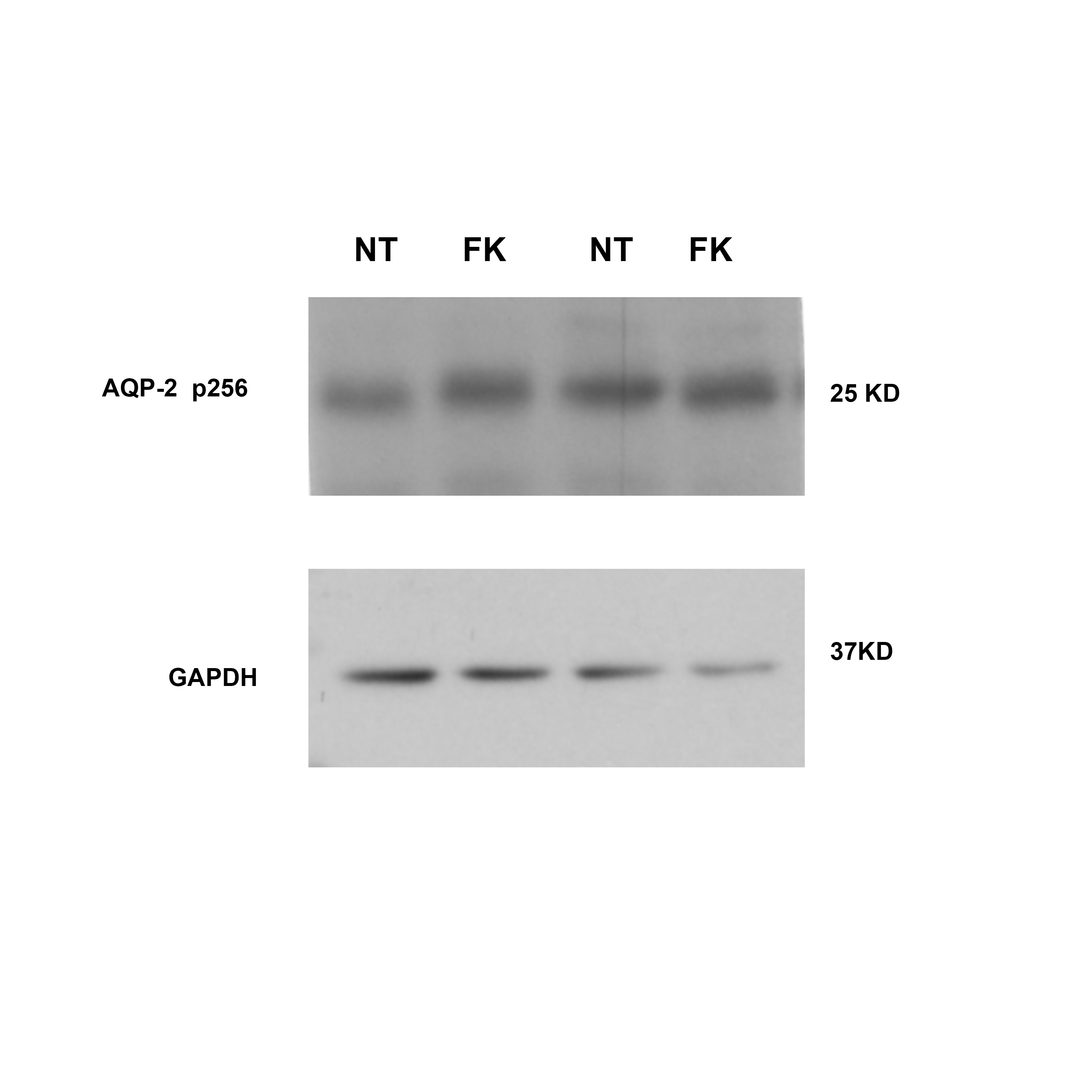

Supplement: S2 Zipfile — These zipfiles contain the additional western blot data used for quantification from stimulated and non-stimulated MDCK cells. The blots are probed with antibodies for GAPDH, total AQP2, as well as pS256, pS261, pS264 and pS269 phospho-AQP2. (ZIP) [file pone.0131719.s004.zip › S4_westerns/AQP2 p256.jpg]

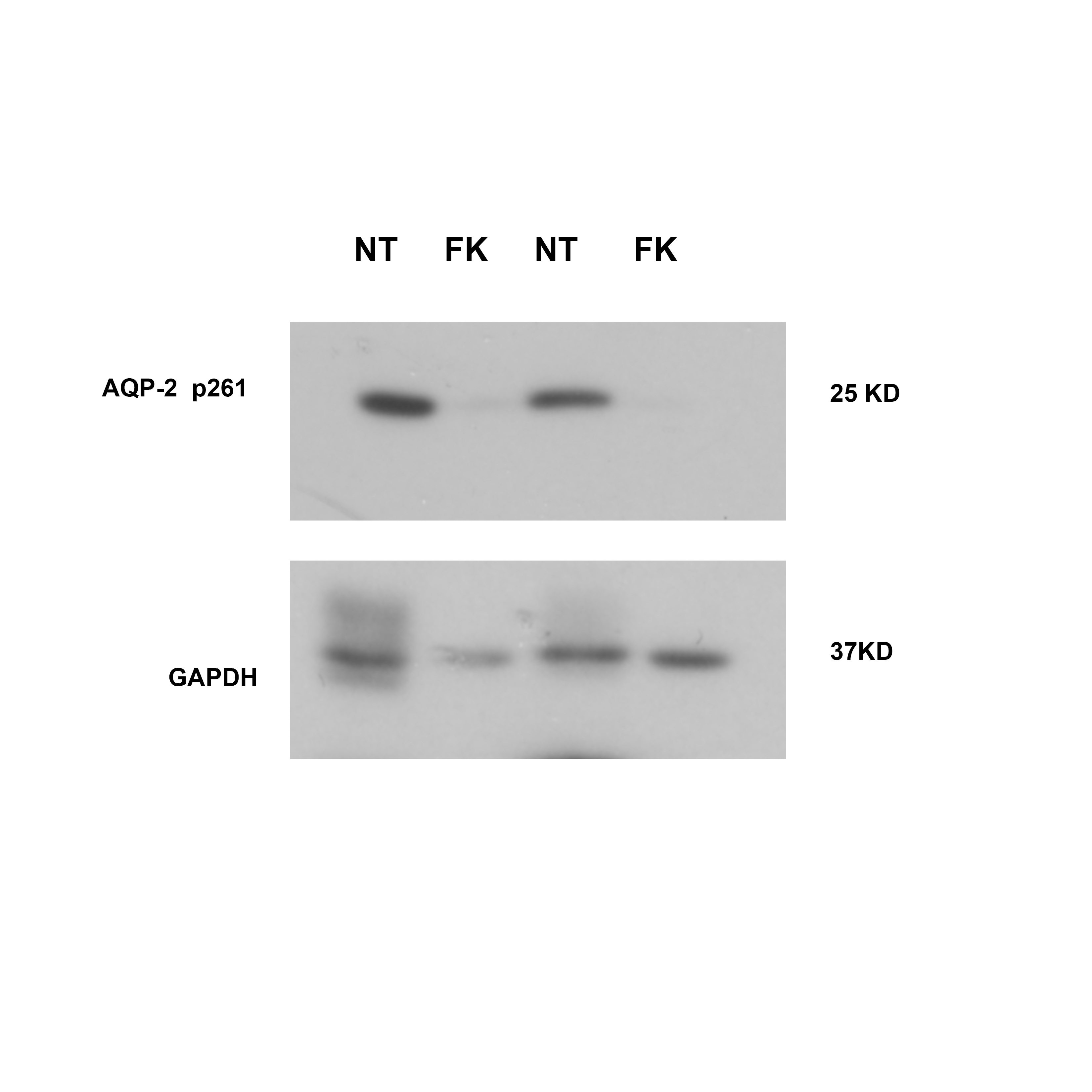

Supplement: S2 Zipfile — These zipfiles contain the additional western blot data used for quantification from stimulated and non-stimulated MDCK cells. The blots are probed with antibodies for GAPDH, total AQP2, as well as pS256, pS261, pS264 and pS269 phospho-AQP2. (ZIP) [file pone.0131719.s004.zip › S4_westerns/AQP2 p261.jpg]

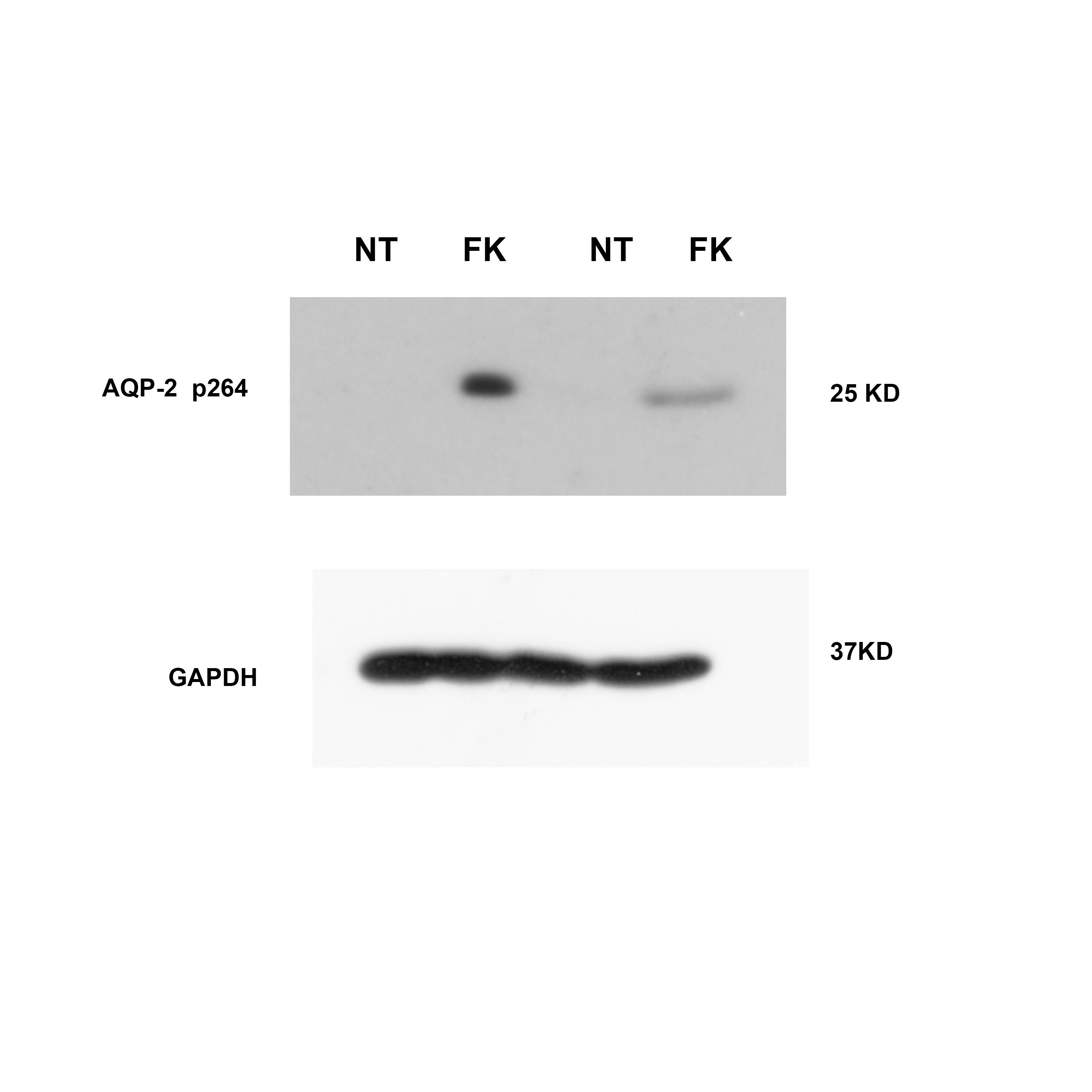

Supplement: S2 Zipfile — These zipfiles contain the additional western blot data used for quantification from stimulated and non-stimulated MDCK cells. The blots are probed with antibodies for GAPDH, total AQP2, as well as pS256, pS261, pS264 and pS269 phospho-AQP2. (ZIP) [file pone.0131719.s004.zip › S4_westerns/AQP2 p264.jpg]

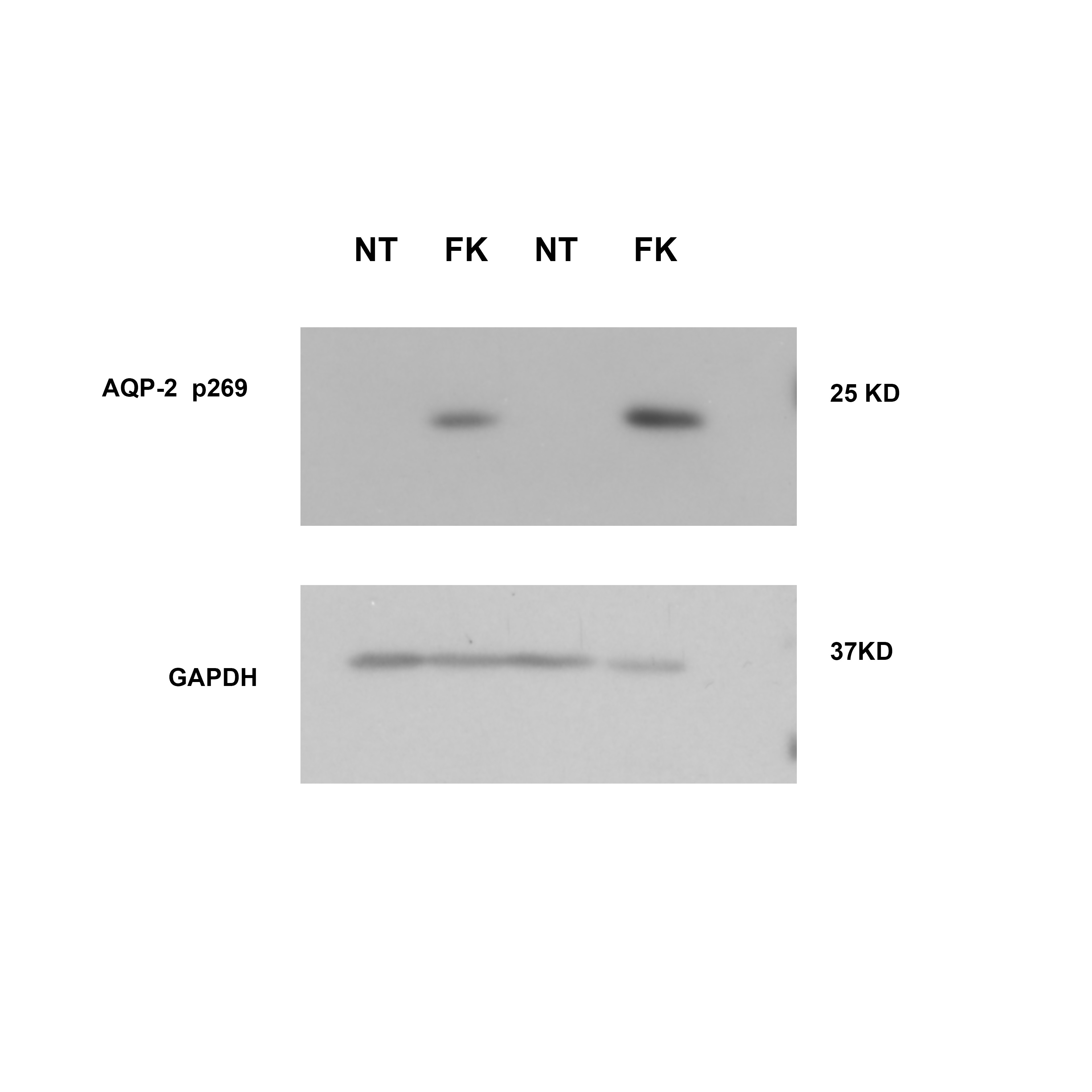

Supplement: S2 Zipfile — These zipfiles contain the additional western blot data used for quantification from stimulated and non-stimulated MDCK cells. The blots are probed with antibodies for GAPDH, total AQP2, as well as pS256, pS261, pS264 and pS269 phospho-AQP2. (ZIP) [file pone.0131719.s004.zip › S4_westerns/AQP2 p269.jpg]

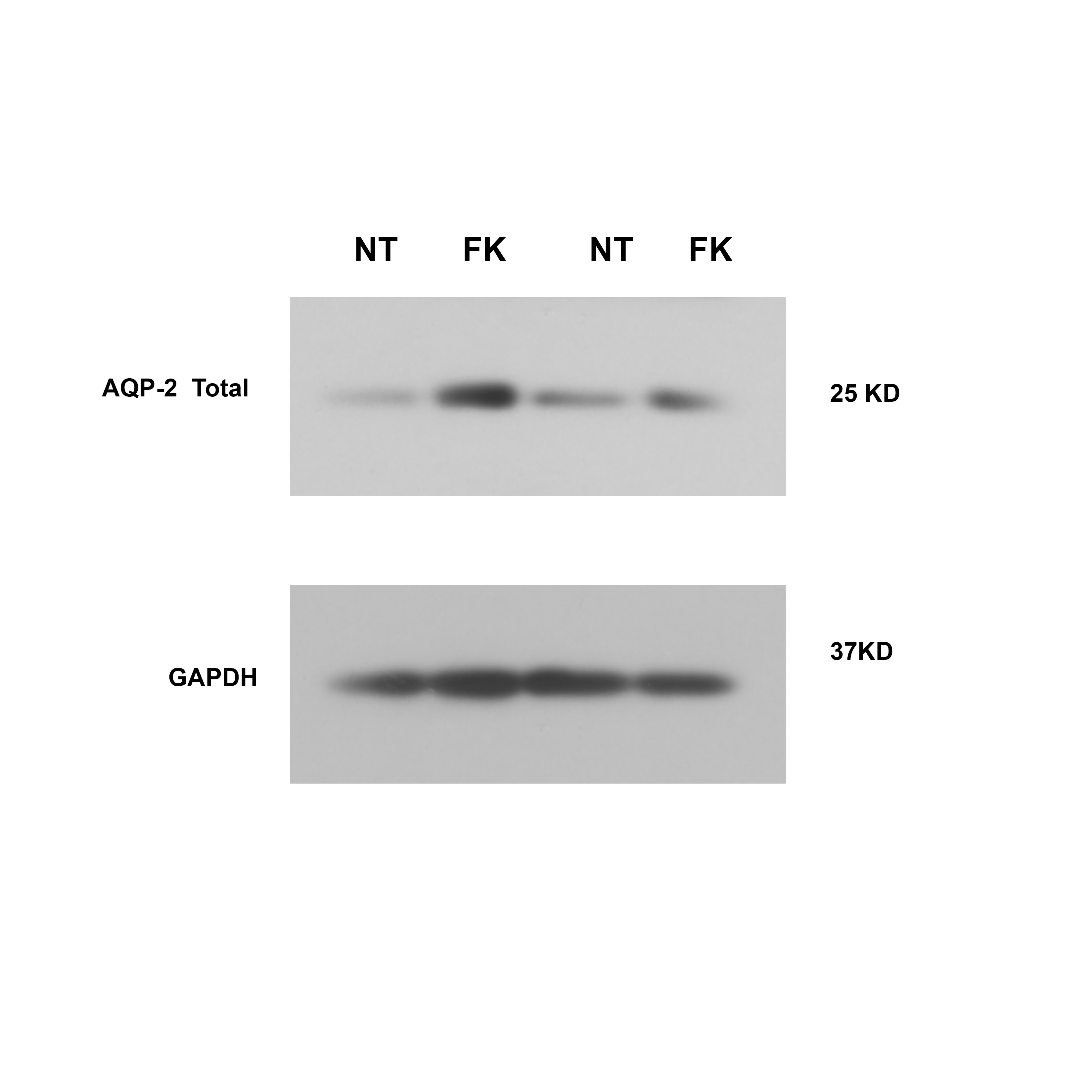

Supplement: S2 Zipfile — These zipfiles contain the additional western blot data used for quantification from stimulated and non-stimulated MDCK cells. The blots are probed with antibodies for GAPDH, total AQP2, as well as pS256, pS261, pS264 and pS269 phospho-AQP2. (ZIP) [file pone.0131719.s004.zip › S4_westerns/AQP2 total.jpg]
